# Weak Capacitance Detection Circuit of Micro-Hemispherical Gyroscope Based on Common-Mode Feedback Fusion Modulation and Demodulation

**DOI:** 10.3390/mi14061161

**Published:** 2023-05-31

**Authors:** Xiaoyang Zhang, Pinghua Li, Xuye Zhuang, Yunlong Sheng, Jinghao Liu, Zhongfeng Gao, Zhiyu Yu

**Affiliations:** School of Mechanical Engineering, Shandong University of Technology, Zibo 255000, China; zxyjiayyou@163.com (X.Z.);

**Keywords:** hemispherical resonant gyro, common-mode feedback, micro-capacitor detection, low noise

## Abstract

As an effective capacitance signal produced by a micro-hemisphere gyro is usually below the pF level, and the capacitance reading process is susceptible to parasitic capacitance and environmental noise, it is highly difficult to acquire an effective capacitance signal. Reducing and suppressing noise in the gyro capacitance detection circuit is a key means to improve the performance of detecting the weak capacitance generated by MEMS gyros. In this paper, we propose a novel capacitance detection circuit, where three different means are utilized to achieve noise reduction. Firstly, the input common-mode feedback is applied to the circuit to solve the input common-mode voltage drift caused by both parasitic capacitance and gain capacitance. Secondly, a low-noise, high-gain amplifier is used to reduce the equivalent input noise. Thirdly, the modulator–demodulator and filter are introduced to the proposed circuit to effectively mitigate the side effects of noise; thus, the accuracy of capacitance detection can be further improved. The experimental results show that with the input voltage of 6 V, the newly designed circuit produces an output dynamic range of 102 dB and the output voltage noise of 5.69 nV/√Hz, achieving a sensitivity of 12.53 V/pF.

## 1. Introduction

With the characteristics of high reliability, high accuracy and long service life, hemispherical resonant gyroscopes (HRGs) have been widely used in multiple fields, such as aerospace, ship navigation and unmanned flight [1,2]. However, due to their high price, large size and high power consumption, the application of HRGs in civil scenarios is limited. In order to promote the application of hemispherical resonant gyro, researchers have designed Micro-compact hemispherical gyros, which inherits the excellent performance of traditional ones, but with a smaller size and lower cost. However, the hemispheric gyro signal is weak and its performance is seriously affected by external environmental factors such as temperature and humidity [3,4]. Therefore, it has become an important and urgent issue to suppress the noise in the capacitance detection process to enable the acquisition and feedback of weak signals generated by Micro-compact hemispherical gyros [5,6,7].

A hemispherical gyroscope can be equated to a dynamic pair of differentially varying capacitance, and by measuring the change in capacitance, information such as the angle/angular velocity can be measured. The output capacitance change of a gyroscope is typically 10^−18^~10^−12^ F, which requires the capacitance detection circuit to have high sensitivity, low noise and large dynamic range [8]. Designing a low-noise, high-accuracy capacitance detection circuit is an important way to improve the performance of a hemispherical gyroscope [9].

The main methods for detecting weak capacitive signals are voltage follower circuits, trans impedance amplifier circuits and charge amplifier circuits. Voltage follower circuits and trans impedance amplifier circuits are susceptible to parasitic capacitance, which reduces the accuracy of the detection circuit. Charge amplifiers provide a virtual ground, and are insensitive to parasitic capacitance and can achieve high gain and low output impedance. When extracting and detecting useful signals, they are easily drowned in noise such as parasitic capacitance or high-frequency interference signal, resulting in effective capacitance signals which cannot be read out. Wu et al. proposed a CMOS low-noise capacitive readout circuit, using a trans impedance amplifier as the core module of the circuit [10], adopting a correlated double sampling technique to suppress the noise and mismatch of the circuit. The noise and mismatch of the circuit are effectively suppressed. By using the full-differential circuit, the performance of the readout circuit in terms of resolution and dynamic range are improved. The circuit also achieves a capacitance resolution of 1.5 aF/Hz^1/2^ and a dynamic range of over 100 dB, but it is susceptible to parasitic capacitance. Li et al. improved the dynamic performance index of the detection signal [11], which are collected from a hemispherical resonator gyro by introducing a correction network and system transfer function. However, a high-input buffer amplifier with impedance is selected for the preamplifier circuit, which is prone to thermal noise and sensitive to the drift of the common-mode voltage.

In order to improve the signal-to-noise ratio, reduce the effects of thermal noise and drift of common-mode voltage [12], in this paper, we employ a charge amplifier to reduce noise and improve the signal-to-noise ratio. In addition, common-mode feedback technology is applied to reduce the interference signal of the operational amplifier circuit, leading to the improvement in extracting and detecting useful signals. Thus, the accurate detection of the capacitor signal produced by hemispherical resonant gyro can be achieved. Moreover, modulation–demodulation and filtering technology are used to improve the accuracy and reduce the noise of the detection circuit.

This paper is conducted in the context of MEMS gyroscope. The article designed a Micro-hemispherical resonator gyro to carry out the experiment, so the method we propose is suitable for MEMS gyro. The research proposes a capacitance detection circuit, where three different means are utilized to achieve noise reduction, including input common-mode feedback, a low-noise high-gain amplifier, and proper modulator–demodulator and filter.

## 2. Device Architecture of Capacitive Displacement Detection

**Figure 1:**
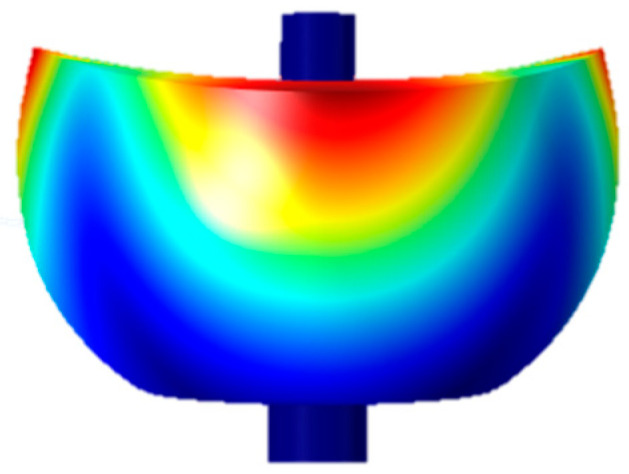
The finite element model of the hemispherical resonator.

**Figure 2:**
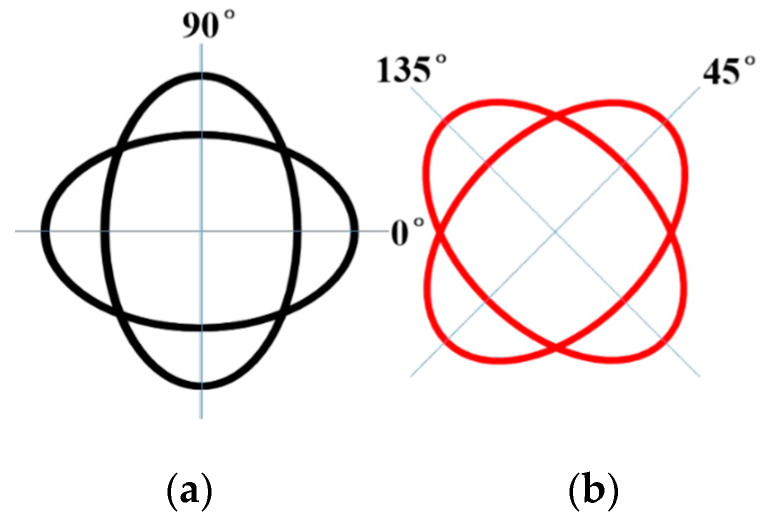
Driving mode and detection mode of miniature hemispherical gyroscope: (**a**) driving mode (**b**) detection mode.

The operating mode of a hemispherical resonant gyroscope resonator is a four-antinode vibration mode (i.e., the number of waves in the annulus m = 2), and the finite element model of the hemispherical resonator is shown in Figure 1 [13]. The mode is synthesized from two simple merged modes, both 45° apart in the direction of the antinode axis, which are the driving and detecting modes of the hemispherical gyro, as shown in Figure 2a,b.

**Figure 3:**
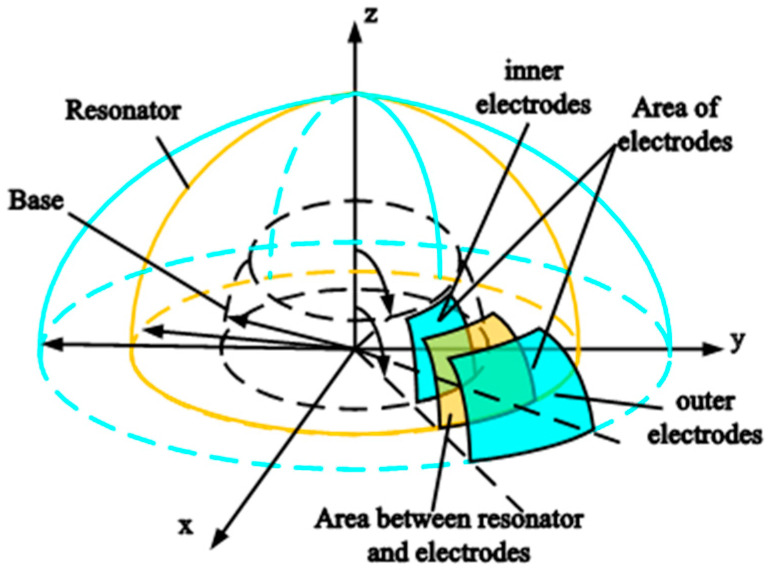
Schematic of detecting capacitance.

The change in capacitance between the resonator shell and electrodes can be described by a schematic of detecting capacitance as shown in Figure 3. The differential capacitance consists of the hemispherical metal resonator, inner and outer detection electrodes distributed on the base. Moreover, variations in capacitance can be calculated accurately by calculating change in capacitance gap.

**Figure 4:**
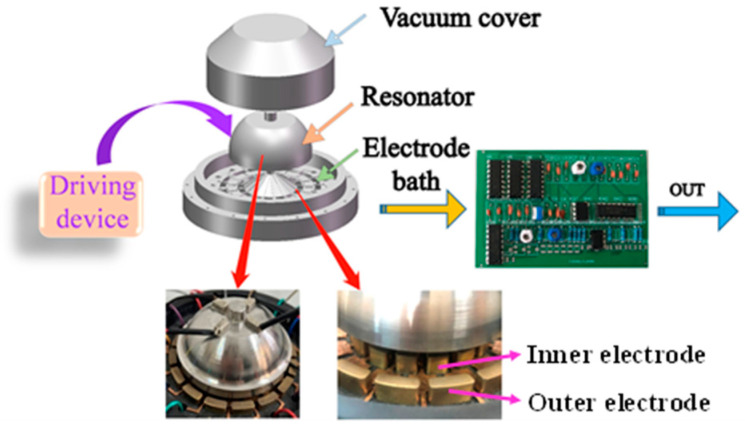
Schematic diagram of excitation and detection of hemispheric resonant gyroscope.

The principle of hemispherical resonator excitation and detection is shown in Figure 4, where the change in capacitance is converted into a voltage (V) by a test circuit, which in turn yields an angular velocity ω. When the hemispherical resonator of the gyroscope vibrates, the detection mode is excited by the Coriolis force, and the amplitude of the resonator detection mode is proportional to the magnitude of the input angular velocity, which is then detected by the detection electrode, and the signal is demodulated to obtain the input angular velocity. The amplitude of the detected mode is proportional to the input angular velocity.

By applying an excitation signal to the resonator through the piezoelectric drive, the resonator will resonate, forming a four-antinode vibration, and the spacing between the resonator and electrode will change. So, a differential will be formed between the electrode and resonator, then a front-end amplifier circuit is connected from the chassis of the gyro structure to detect the capacitance change (∆C). The differential electrode structure is shown in Figure 4, and the capacitance changes will be transformed by the test circuit into a voltage (V), which in turn gives the angular velocity (ω).

## 3. Circuit Design

### 3.1. Noise Analysis

**Figure 5:**
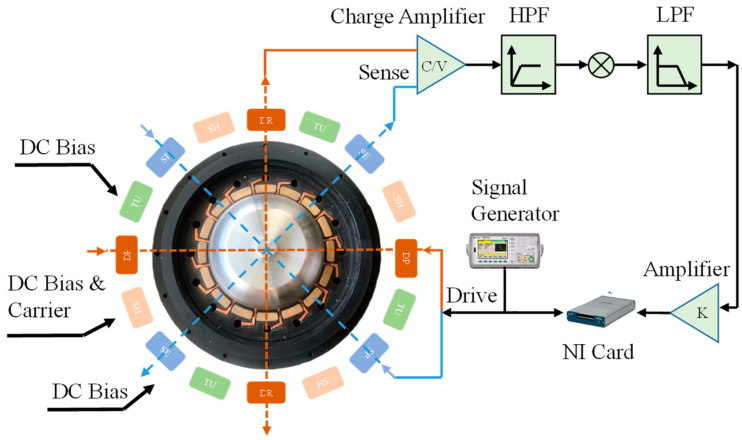
Block diagram of the hemispheric resonant gyroscope detection system.

Because of the small amplitude of the resonators’ vibration, the gyro output signal is very weak. The readout circuit requirements are strict on noise, sensitivity and the compatibility of devices. The noises affecting the accuracy of the circuit are mainly the following: (1) thermal noise of the input MOSFET and feedback resistor; (2) 1/f noise of the input MOSFET; (3) reference noise generated by the reference power supply $V_{ref}$; (4) shot particle noise generated by the reverse bias diode leakage current. Figure 5 shows the block diagram of the Micro-miniature hemispherical resonant gyroscope detection circuit. The electrodes of different phases are excited by the piezoelectric drive, and a weak signal is generated. After, through the front-end amplifier circuit and C/V conversion circuit, the effective signal is obtained with a filter. Then, the effective signal is returned to the driving mode through the NI card and signal generator, thus forming a closed loop [14,15,16].

The output voltage of the detection circuit charge amplifier *V_F_* is

(1) $$V_{F}=\Delta V\left(\frac{\partial C_{1}}{\partial t}-\frac{\partial C_{2}}{\partial t}\right)\times \frac{1}{C_{fp}s+1/R_{fp}}$$

**Figure 6:**
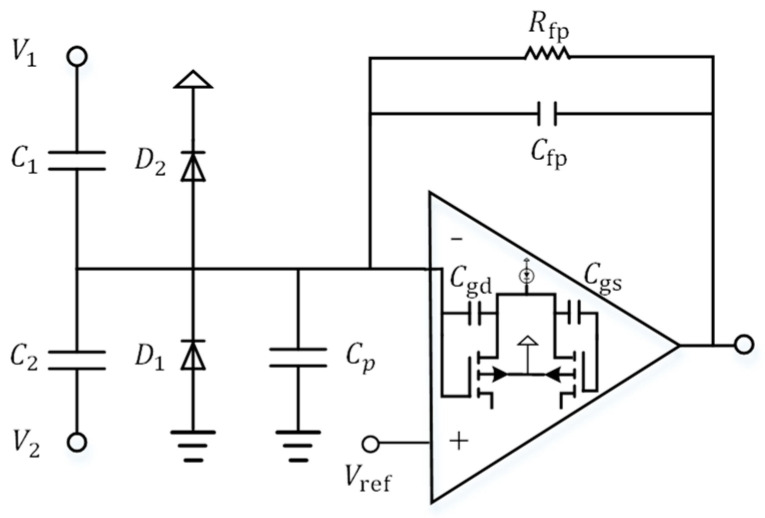
The parasitic capacitance structure of the detection circuit of the charge amplifier.

After the inverting terminal of the charge amplifier reaches the new negative reference voltage, *V_ref_* is at the non-inverting end, the charge amplifier changes state again and the output is driven to the opposite supply rail voltage + V (sat). The capacitor gains a positive voltage across its plate and the charging cycle begins again. Thus, the capacitor is constantly charged and discharged, thus stabilizing the output of the charge amplifier signal. So, the charge amplifier circuit can reduce the effect of parasitic capacitance “*C_p_*” at the output of the amplifier by using “*V_ref_*” at the non-inverting terminal. For charge amplifiers, including differential amplifiers, there are non-inverting terminals connected to the reference voltage (*V_ref_*) “+”and inverting terminals “−”connected to the output of the amplifier via a feedback capacitor (*C_fp_*). The structure of the parasitic capacitance of the charge amplifier detection circuit is shown in Figure 6, where *C*_1_ and *C*_2_ are the capacitance of the gyroscope detect port, which are both *C*; *C_p_* is the total parasitic capacitance of the circuit input, which mainly consists of the MOSFET gate capacitance *C_gd_* and *C_gs_*; *R_fp_* and *C_fp_* are the integral capacitance and the feedback resistor of the charge amplifier, respectively.

The reference voltage is *V_ref_*, half of the voltage value of the power supply, and *V_O_*_1_ is the generated noise. The reference voltage noise at the output of the op-amp *V_O_*_1_ is

(2) $$V_{O1}=\left(1+\frac{C_{p}+C}{C_{fp}}\right)\times V_{1}$$

The thermal noise, *V_O_*_2_, is generated at the output of the operational amplifier, which can be defined as

(3) $$V_{O2}=\frac{4KTR_{fp}}{2\pi fR_{fp}C_{fp}+1}$$

The shot noise generated by the charge amplifier *V_amp_* can be written as

(4) $$V_{amp}=\frac{4\mathrm{KT}}{\sqrt{2\mu C_{\mathrm{OX}}\left(W/L\right)I_{D}}}+\frac{k}{C_{\mathrm{OX}}\mathrm{WL}}\times \frac{1}{f}$$

where W and L are the channel width and length parameters entered into the MOSFET, I_D_ is its bias current, K is the Boltzmann constant, T is the absolute temperature, µ is the carrier mobility, C_OX_ is the gate capacitance per unit area, k is the flicker noise factor and *f* is the frequency of circuit operating.

The shot noise at the output of the charge amplifier *V_O_*_3_ can be described as

(5) $$V_{O3}=\left(1+\frac{C_{p}+C}{C_{fp}}\right)\times \frac{4\mathrm{KT}}{\sqrt{{2\mu C}_{\mathrm{OX}}\left(W/L\right)I_{D}}}+\frac{k}{C_{\mathrm{OX}}\mathrm{WL}}\times \frac{1}{f}$$

The total output noise of the charge amplifier *V_out_* is analyzed by the above calculation:

(6) $$V_{out}=\left(1+\frac{C_{p}+C}{C_{fp}}\right)V_{1}+\frac{4\mathrm{KT}R_{fp}}{2\pi fR_{fp}C_{fp}+1}+\left(1+\frac{C_{p}+C}{C_{fp}}\right)\frac{4\mathrm{KT}}{\sqrt{{2\mu C}_{\mathrm{OX}}\left(W/L\right)I_{D}}}+\frac{k}{C_{\mathrm{OX}}\mathrm{WL}}\frac{1}{f}$$

It shows clearly in Equation (6) that the output noise of charge amplifier is mainly affected by thermal noise and scintillation noise. At low frequencies, the circuit is affected by flicker noise. When the circuit operating frequency rises, the noise decays rapidly. The resonant frequency of the gyro is 6 kHz, and the noise level can be simplified as follows:

(7) $$V_{out}=\left(1+\frac{C_{p}+C}{C_{fp}}\right)\times V_{1}+\left(1+\frac{C_{p}+C}{C_{fp}}\right)\times \frac{4\mathrm{KT}}{\sqrt{{2\mu C}_{\mathrm{OX}}\left(W/L\right)I_{D}}}$$

As can be seen from Equation (7), *V_out_* ∝ 1/*C_fb_* when increasing the feedback capacitance of the charge amplifier, the noise at the output of the circuit will gradually decrease. However, as can be seen from Equation (1), *V_F_* ∝ 1/*C_fb_* increasing *C_fb_* will lead to a reduction in the signal-to-noise ratio of the circuit. Therefore, to improve the accuracy of the circuit, the value of *C_fb_* has to be reduced appropriately. In order to obtain a better overall performance of the circuit, the value of *C_fb_* needs to be compromised according to the requirements of the gyroscope’s using environment.

### 3.2. Operational Amplifier Design

**Figure 7:**
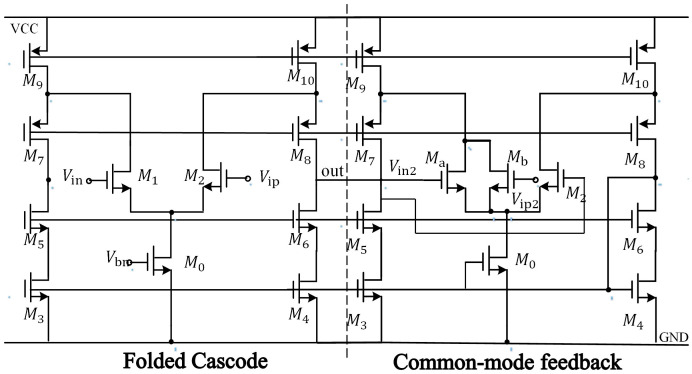
Structure diagram of operational amplifier.

The operational amplifier is the core module of the interface circuit, the noise, mismatch and limited DC gain of the operational amplifier will introduce errors into the capacitor–voltage conversion, resulting in poor detection accuracy [17,18]. Since the first-stage amplifier processes an extremely weak signal with a small output voltage amplitude, it is necessary to pay special attention to low noise and high gain when designing a gyro detection circuit. A folded cascode CMOS operational amplifier structure with PMOS tube input as shown in Figure 7 is used to achieve a large output voltage amplitude and wide dynamic range for the detection circuit; the output stage uses a class AB output structure to achieve a rail-to-rail output. The common-mode feedback compares the reference voltage with the common-mode value at the differential input of the main op-amp. The error value is sampled and delivered to the input feedback capacitor at the second phase, and when the next phase arrives, both pole plates of the input feedback capacitor are connected to the reference voltage. The common-mode feedback circuit introduced at the differential input reduces the parasitic capacitance and stabilizes the input common-mode voltage, while also effectively suppressing the thermal noise caused by the common-mode voltage drift and ensuring the stability of the input common-mode voltage of the main op-amp. Table 1 shows the technical index of the op-amp.

**Figure 8:**
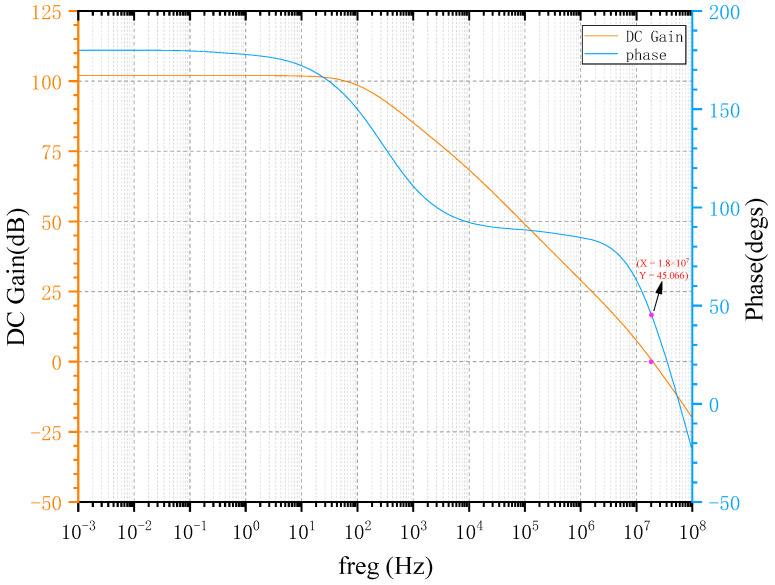
Open-loop amplitude frequency characteristic curve of operational amplifier.

According to the technical specifications of the operational amplifier, and combined with Equations (1) and (7) to analyze and calculate the circuit, the integral capacitance of the charge amplifier is about 3.5 pF. Phase margin is a very important index in circuit design, which is mainly used to measure the stability of the negative feedback system and to predict the overshoot of the step response of the closed-loop system. For a good performance control system, the phase margin should be about 45 degrees. An amplifier with a small phase margin will respond longer, while an amplifier with a larger phase margin will take longer to rise to the final level of the voltage step, so the phase margin is about 45 degrees. The obtained open-loop amplitude and phase frequency curve of the amplifier are shown in Figure 8. Among them, the open-loop unit gain bandwidth of the operational amplifier is about 18 MHz and the open-loop gain is about 102 dB.

## 4. Modulation–Demodulation Techniques

**Figure 9:**
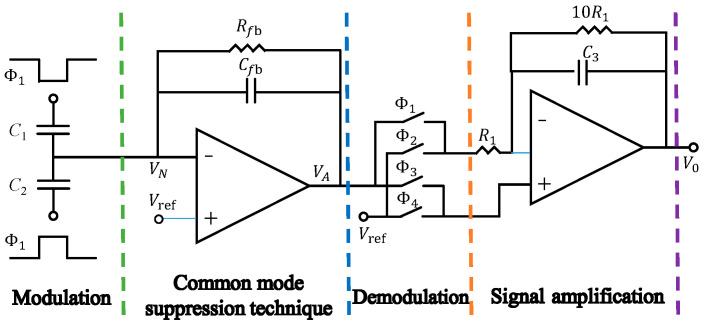
Design of gyroscope capacitance readout circuit.

The modulation/demodulation technique is to modulate the current signal generated by the gyroscope via the stimulus signal. Specifically, it uses the dichotomous frequency relationship between the stimulus signal and the gyroscopic angular velocity signal to selectively amplify the signal. Using the filtering technique to process the signal of the first-stage amplification circuit, the band-pass filter outputs the frequency-doubled signal, retains the amplified angular velocity signal, and outputs it through the second-stage amplification circuit to complete the whole signal processing. Equation (7) shows that the circuit noise and frequency are inversely proportional to each other. When the frequency increases, the output noise of the circuit is reduced. Based on the characteristic of low noise in high frequency of the circuit, the modulation/demodulation technology could separate the signal from the noise and amplify an effective signal. Such operations effectively improve the accuracy of the readout circuit. The framework of the designed readout circuit for gyroscope capacitance is shown in Figure 9; the carrier signals are φ_1_ and φ_2_, and the demodulation switch is ∅1,∅2,∅3 and ∅4. The current signal generated by MEMS gyroscope is modulated by carrier signal, and the signal is selectively amplified by using the different working frequencies of carrier signal and gyroscope angular velocity signal, and the signal is processed by reasonable demodulation technology.

Assume that the angular rate of gyroscopic detection is Ω and its magnitude is equal to

(8) $$\Omega =\cos\left(\omega_{\Omega }t\right)$$

where *ω*_Ω_ is the rate of change in angular velocity.

The modulation of the small signal generated by the gyroscope with a carrier signal value of

(9) $$f_{n}=\cos\left(\omega_{n}t\right)$$

where *ω_d_* is the frequency of the carrier signal, then the output of the charge amplifier at the detection end is

(10) $$V_{A}\infty 2\times \Omega \times V\times f_{n}=2\cos\left(\omega_{\Omega }t\right)\times \cos\left(\omega_{d}t\right)\times \cos\left(\omega_{n}t\right)$$

The signal is demodulated in two steps, starting with the carrier signal cos (*ω_n_t*) and then the drive signal cos (*ω_d_t*). The high-frequency signal is filtered using a filtering technique to obtain an output voltage proportional to the low-frequency angular velocity signal.

(11) $$V_{0}\infty \cos\left(\omega_{\Omega }t\right)$$

## 5. Analysis of Test Results

**Figure 10:**
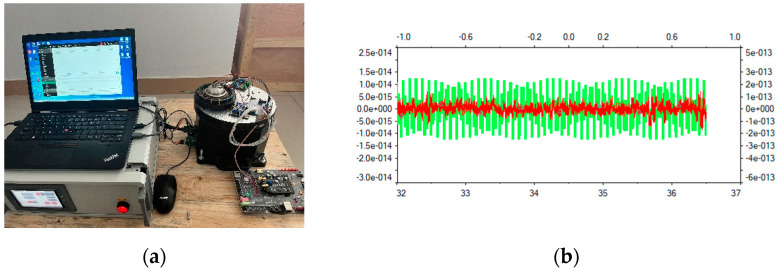
Transient response test diagram of readout circuit: (**a**) the experimental test set-up (**b**) the read channel’s transient response.

Using a sine signal of a certain amplitude generated by the signal generator as input, the waveforms of the positive and negative outputs of the circuit can be measured with an oscilloscope. The experimental test set-up is shown in Figure 10a, and the results of the read channel’s transient response are shown in Figure 10b. In Figure 10b, the red curve represents the output voltage of the circuit before the improvement, from which it can be seen that the signal is messy and the noise is not suppressed. By using the methods of this research, the waveform changes from a messy 0.5 V to a 1 V sine wave. The green curve is acquired with the improved circuit, where the signal is sinusoidal, and the noise is effectively suppressed without obvious distortion.

**Figure 11:**
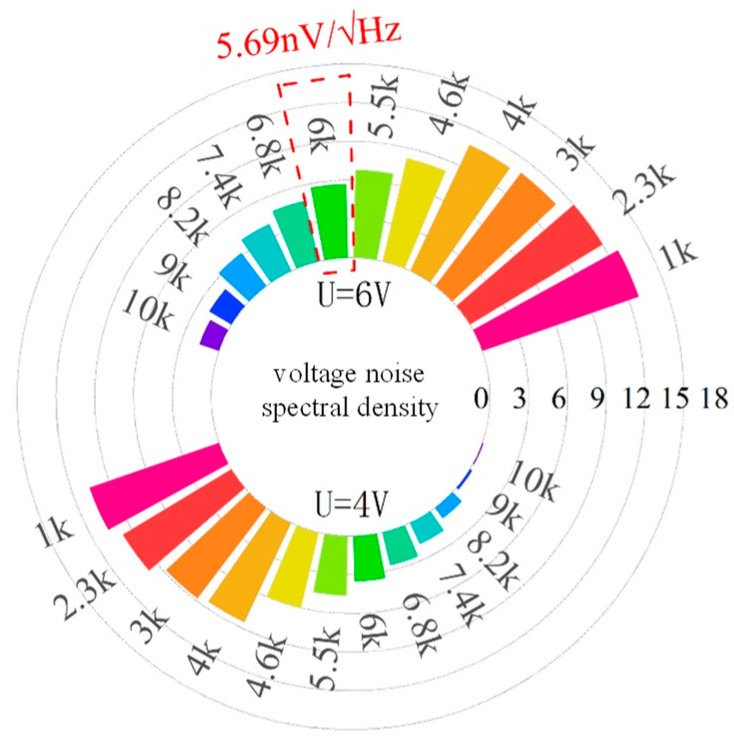
The spectrum density simulation diagram of voltage noise.

**Figure 12:**
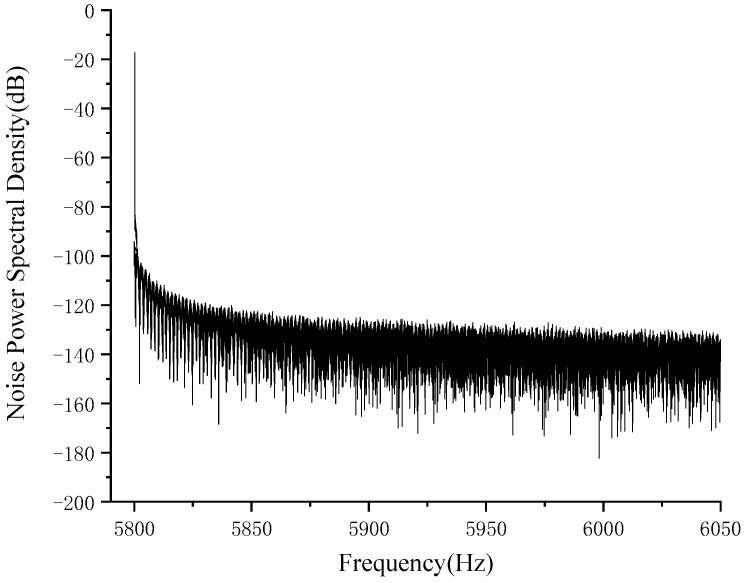
The noise power spectral density of the amplifier output noise.

The noise of the readout circuit is obtained using simulation for the voltage noise spectral density curve. The resonant frequency of MEMS gyroscope is 6 KHz, and the applied voltage is 4 V and 6 V, where the output voltage noise is 5.69 nV/√Hz and the readout circuit noise ratio is approximately 0.27 in Figure 11. Then, the output noise of the amplifier designed in this article is carried out to obtain the noise power spectral density graph. The resonant frequency of gyro is close to 6 kHz, and the noise power spectral density is about −63.34 dBVrms/rtHz. The noise power spectral density of the amplifier output noise is shown in Figure 12.

**Figure 13:**
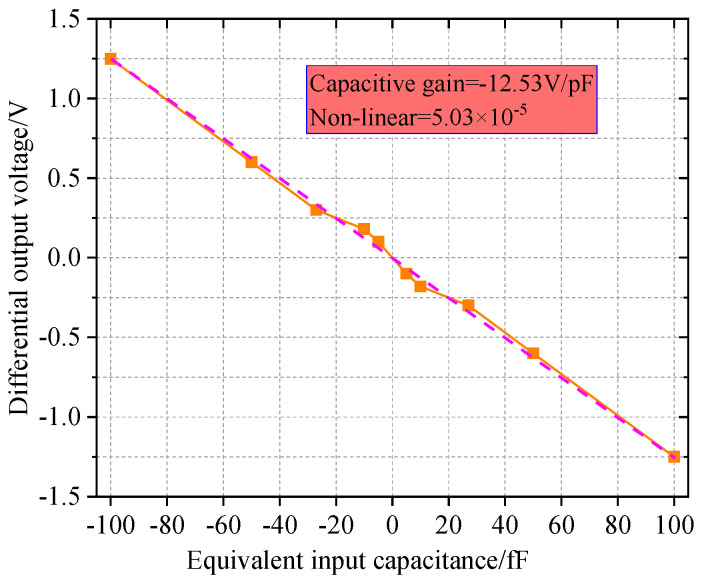
Sensitivity curve of input capacitance and output voltage.

The relationship among the circuit sensitivity, capacitance and voltage can be described in Figure 13. When the differential capacitance of gyroscope changes, the operational amplifier can output 1~3 V voltage. As the capacitance increases from −100 fF to + 100 fF, the orange lines represent 10 experiments and 10 sets of data, and the red lines represent error lines, the sensitivity of the circuit maintains at 12.53 V/pF, the nonlinearity is 5.03 × 10^−5^ and the operational amplifier circuit output voltage is 1.25 V. The conclusion is consistent with the technical index of the operational amplifier mentioned above.

**Figure 14:**
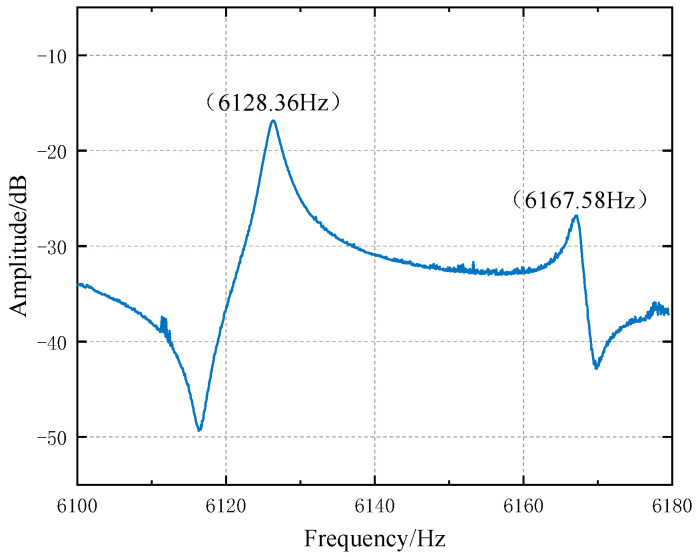
Frequency response of the resonant gyroscope.

In order to verify the accuracy of the circuit, we designed a small metal gyroscope; the frequency of the small metal gyro is 6 kHz with the biggest capacitance change of 0.5 pF. Figure 14 shows the measurement of frequency sweep for the four-antinode vibration mode tested at room temperature using network analyzer. Through the circuit-to-sweep frequency, the resonant frequency of the resonator is 6.12836 kHz with a quality factor of 2665. The results are consistent with the designed gyro frequency; it is proven that the circuit can be used as the weak capacitance detection circuit of MEMS gyroscope. Although the correctness of the circuit is verified, this method is complicated, and the process of testing requires a quiet space along with avoiding the introduction of any new noise.

**Figure 15:**
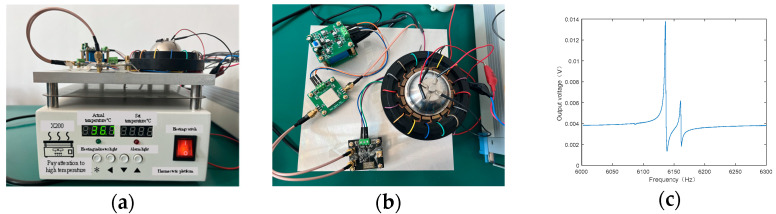
The long-term stability test of the readout circuit: (**a**) the experimental test set-up at different temperatures; (**b**) top view of the experimental test set-up; (**c**) frequency response of the resonant gyroscope.

In order to verify the long-term stability of the circuit, the designed circuit boards were tested at 20 degrees Celsius, 30 degrees Celsius and 40 degrees Celsius in Figure 15. At different temperatures, the test results are consistent with those in Figure 14, and the resonant frequency is 6.128 kHz. The test results show that the output signal of the circuit has no obvious change, which verifies the long-term stability of the design.

The comparisons of the noise performance between different weak capacitance readout circuits are shown in Table 2. The circuit proposed in this article adopts common-mode feedback technology and modulation and demodulation technology, obtaining the output voltage noise of 5.69 nV/√Hz and sensitivity of 12.53 V/pF. Wu et al. proposed a CMOS low-noise capacitive readout circuit, using a trans impedance amplifier as the core module of the circuit [10], adopting a correlated double sampling technique to suppress the noise and mismatch of the circuit. The noise and mismatch of the circuit are effectively suppressed. By using the full-differential circuit, the performance of the readout circuit in terms of resolution and dynamic range are improved. The circuit also achieves a capacitance resolution of 1.5 aF/Hz^1/2^ and a dynamic range of over 100 dB, but it is susceptible to parasitic capacitance. Compared with reference [10] (1.5 aF/√Hz) and reference [19] (1.7 aF/√Hz), the capacitance resolution of the designed circuit is 0.1 aF/√Hz, which is improved by an order of magnitude. The sensitivity of the circuit introduced by reference [20] is 12.58 V/pF, which is slightly bigger than that of the circuit proposed in this article. In Ref. [17], an operational amplifier with a high power rejection ratio and low noise was designed, and the output level noise floor of the capacitive readout circuit was −117.14 dB, but the preamplifier circuit is chosen to be a transoresistor amplifier, which is easy to produce thermal noise and sensitive to the drift of the common-mode voltage. The output voltage noise ratio of our circuit is higher than the Northern General Electronics Group Limited circuit [16], which is increased by a factor of 0.27. The circuit designed here has the characteristics of low noise, small capacitive resolution and high sensitivity, which can be used in the field of weak capacitance detection.

## 6. Conclusions

With benefits including high precision, high reliability, long life and simple structure, the gyroscope has been widely used in many fields such as aerospace, autonomous vehicles and robotics. The rapid development of micromachines has put forward higher and higher requirements for the performance of subsequent readout circuits. However, the development of a high quality resonator does not make a high performance gyroscope. The interface and control systems circuit must be carefully designed to take full advantage of the resonator’s capabilities and compensate for its non-idealities. The capacitance produced by the gyroscope changes very little. The weak capacitance changes are easily submerged in noise. Designing a weak capacitance detection circuit is of great significance to improve the performance of a gyroscope. The design and implementation of the electronic control and readout interface circuit is just as important as that of the resonator for defining the final performance of the gyroscope.

A weak capacitance detection circuit is designed to detect the capacitance generated by hemisphere gyro. In the circuit, the input common-mode voltage drift, which is caused by parasitic and gain capacitance, is effectively reduced via input common-mode feedback. A low-noise high-gain amplifier is applied to further suppress the equivalent input noise. Furthermore, the accuracy of the proposed circuit is significantly improved by modulator–demodulator and filter techniques, which work well in combating noise. Experimental results show that the output voltage noise, sensitivity, voltage and dynamic range of the proposed circuit are 5.69 nV/√Hz, 12.53 V/pF, 1.25 V and 102 dB, respectively. Such results demonstrate the effectiveness of the proposed circuit and verify that it can be used in the weak capacitance detection of Micro-hemisphere gyroscopes, and it provides a useful research value for the rapid development of micromachinery.

## Figures and Tables

**Table 1:** Technical index of operational amplifiers.

Performance Parameters	Indicators	Unit
Open-loop gain	102	dB
Gain bandwidth	18	MHz
Phase margin	45	°
Supply voltage	6	V
Equivalent input noise	6	nV/√Hz
Output amplitude	1~3	V

**Table 2:** Performance comparison of different weak capacitor readout circuits.

	Output Voltage Noise (nV/√Hz)	Capacitive Resolution (aF/√Hz)	Sensitivity (V/pF)	Remarks
Institute of Electronics, Chinese Academy of Sciences	/	1.5	16.7	Reference [10]
East China Institute of Optoelectronic Integrated Devices	/	0.06	12.58	Reference [20]
Northern General Electronics Group Limited	7.82	0.116	/	Reference [16]
Amirkabir University of Technology	/	1.7	/	Reference [19]
This article	5.69	0.1	12.53	
